# Specific Association of Lectin LecB with the Surface of *Pseudomonas aeruginosa*: Role of Outer Membrane Protein OprF

**DOI:** 10.1371/journal.pone.0046857

**Published:** 2012-10-08

**Authors:** Horst Funken, Kai-Malte Bartels, Susanne Wilhelm, Melanie Brocker, Michael Bott, Manjeet Bains, Robert E. W. Hancock, Frank Rosenau, Karl-Erich Jaeger

**Affiliations:** 1 Institute of Molecular Enzyme Technology, Heinrich-Heine-University Duesseldorf, Juelich, Germany; 2 Institute of Bio- and Geoscience 1, Forschungszentrum Jülich, Juelich, Germany; 3 Department of Microbiology and Immunology, University of British Columbia, Vancouver, Canada; 4 Institute of Pharmaceutical Biotechnology, Ulm-University, Ulm, Germany; Vrije Universiteit Brussel, Belgium

## Abstract

The fucose binding lectin LecB affects biofilm formation and is involved in pathogenicity of *Pseudomonas aeruginosa.* LecB resides in the outer membrane and can be released specifically by treatment of an outer membrane fraction with fucose suggesting that it binds to specific ligands. Here, we report that LecB binds to the outer membrane protein OprF. In an OprF-deficient *P. aeruginosa* mutant, LecB is no longer detectable in the membrane but instead in the culture supernatant indicating a specific interaction between LecB and OprF.

## Introduction

Lectins are proteins of non-immune origin that recognize and bind to specific carbohydrate structural epitopes. This group of carbohydrate-binding proteins function as central mediators of information transfer in biological systems and perform their duties by interacting with glycoproteins, glycolipids and oligosaccharides [1]. They are found in a wide range of organisms including viruses, bacteria, plants and animals, and are believed to play an important role in cell-cell interactions [2]. Bacteria possess several different types of lectins [3], including for example FimH which is located at the top of type 1 pili from the uropathogenic *Escherichia coli* and recognizes terminally located D-mannose moieties on cell-bound glycoproteins mediating adhesion between the bacterium and the urothelium [4], [5]. Furthermore, lectins may have a significant biotechnological and medical potential, as exemplified by the galactoside-specific mistletoe lectin, which is used on a large scale to support anti-cancer therapy [6].

*P. aeruginosa*, an opportunistic pathogen associated with chronic airway infections, synthesizes two lectins LecA and LecB (formerly named PA-IL and PA-IIL) [7]. Strains of *P. aeruginosa* that produces high levels of these virulence factors exhibit an increased virulence potential [8]. Both lectins play a prominent role in human infections, since it was demonstrated that *P. aeruginosa*-induced otitis externa diffusa [9], as well as respiratory tract infections [10] including those in cystic fibrosis (CF) patients [11], could be successfully treated by application of a solution containing LecA and LecB- specific sugars. The sugar solutions presumably prevented the lectin-mediated bacterial adhesion to the corresponding host cells.

The expression of lectin genes in *P. aeruginosa* is coordinately regulated with certain other virulence factors and controlled via quorum sensing and by the alternative sigma factor RpoS [12]. LecB consists of four 11.73 kDa subunits, each exhibiting a high binding constant for L-fucose (K_D_ = 1.5×10^6^ M^−1^) and its derivatives [13], [14] and a somewhat lower binding constant for D-mannose (K_D_ = 3.1×10^2^ M^−1^). The crystal structure of LecB purified from *E. coli* showed a tetrameric organisation of the protein stabilized by Ca-ions with four sugar binding sites each composed of residues from two subunits [15], [16], [17]. Recently, we have demonstrated the N-glycosylation of LecB which appears to be required for proper transport to its final destination on the cell surface of *P. aeruginosa* [14].

In CF patients, increased terminal fucosylation of airway epithelial glycoproteins is found, as well as a higher percentage of sialylated and sulfated oligosaccharides in Lewis A oligosaccharide side chains, which presumably represent preferential ligands for LecB [16] thereby contributing significantly to chronic respiratory *P. aeruginosa* infections [18]. Interestingly, LecA and LecB also inhibit ciliary beating [19] which represents an important defence mechanism of the lung [20], [21]. It was suggested that LecB is exposed on the surface of sessile *P. aeruginosa* cells, since the addition of L-fucose-branched chitosan led to specific cell aggregation [22]. In addition, it was shown that LecB is located in the bacterial outer membrane and a *lecB*-deficient *P. aeruginosa* strain is impaired in biofilm formation [23]. Addition of glycopeptide dendrimers targeting LecB resulted in complete inhibition and dispersion of biofilms, which clearly marks this lectin as a valuable target for developing *P. aeruginosa* biofilm inhibitors [24], [25]. LecB is also involved in the assembly of pili on the cell surface and in the production protease IV [26].

Cell surface appendages of *P. aeruginosa*, like pilus and flagella function as adhesins that bind to receptors, e.g. those present on the respiratory epithelium, thus initiating bacterial adherence [27], [28], [29]. The outer membrane protein OprF has been identified as an adhesin for human alveolar epithelial (A549) cells [30]. OprF is a major outer membrane porin forming a non-specific, weakly cation-selective channel with two different channel sizes [31], [32], [33]. Interestingly, full length OprF is required for the formation of large pores whereas C-terminal truncations only form smaller sized pores [34] suggesting that OprF can adopt different conformations [35]. Furthermore, OprF plays an important role in antimicrobial drug resistance and has also been suggested as a vaccine component [36]. Gene disruption and gene deletion analyses have indicated that it is also required for cell growth in low-osmolarity medium, the maintenance of cell shape and peptidoglycan association [35].

In this paper we report that LecB is exposed on the surface of sessile *P. aeruginosa* cells where it interacts with the outer membrane porin OprF. Treatment of biofilm cells with L-fucose resulted in the release of LecB, whereas treatment with D-galactose had no effect. The interaction of LecB with OprF was directly demonstrated using N-terminal His-tagged LecB immobilized on Ni-NTA agarose and by affinity chromatography on a mannose agarose column, which resulted in co-purification of LecB and OprF. We furthermore observed that an OprF-deficient *P. aeruginosa* mutant secretes LecB into the culture medium indicating that this lectin binds to OprF on the bacterial cell surface.

## Experimental Procedures

### Animals and Ethics Statement

Animal blood used in this study was purchased from Fiebig Nährstofftechnik (Idstein-Niederauroff/Ts, Germany). The company is licensed to produce animal blood samples for diagnostic and biotechnology use by the regional council of Darmstadt (Germany), in accordance to chapter IV article 18 Abs. 1 of decree (EG) Nr. 1774/2002 under control of veterinary control number DE 06 439 0001 14.

### Bacterial Strains and Plasmids

The strains and plasmids used in this study are listed in Table 1. *E. coli* was used for cloning experiments and *E. coli* BL21(DE3) as a heterologous expression host for plasmid encoded LecB. *E. coli* S-17 was used for conjugal transfer.

**Table 1:** Bacterial strains and plasmids.

Strain/Plasmid	Genotype/Phenotype	Reference/source
**Strains**		
***P. aeruginosa***		
PAO1	wilde-type	60
PATI2	LecB mutant strain derived from PAO1. *lecB::Gmr*	23
H636	OprF mutant strain derived from PAO1. *oprF::Strepr*	35
***E. coli***		
BL21(DE3)	*F2 ompT hsdSB(rB 2 mB 2 ) gal dcm (lcIts857 ind1 Sam7 nin5 lacUV5-T7 gene1)*	61
S17.1	*Ec294 : : [RP4-2 (Tc : : Mu) (Km : : Tn7)], pro, res, recA, tra+, Tpr , Sm*	62
**Plasmids**		
pEC2	*pET22b containing the 345 bp NdeI/BamHI PCR product with the lecB gene*	15
pURE	*pET19 containing the coding regions for a hexahistidin tag, an entrokinase cleavagesite* *and the LecB protein*	37
pBBC2	*pBBR1MCS containing a 398 bp XbaI/SacI fragment with lecB derived from pEC2*	23

### Media and Growth Conditions

Pre-cultures for all experiments were prepared overnight in 10 ml LB medium at 37°C. Plasmid-carrying *E. coli* cells were selected with 100 µg ampicillin ml^−1^ and 50 µg chloramphenicol ml^−1^. In the case of plasmid- or cassette-carrying *P. aeruginosa* strains, 300 µg chloramphenicol ml^−1^, 50 µg gentamicin ml^−1^ and/or 500 µg streptomycin ml^−1^ were added.

*P. aeruginosa* cells were grown as unsaturated biofilms on NB-agar plates for 48 h at 37°C and bacterial cells were isolated by washing with PBS and subsequent centrifugation for 10 min at 3,000×g.

### Overexpression of lecB and lecB::his6

Expression cultures were grown at 37°C in 1 L of LB medium containing 0.4 % (w/v) glucose in 5 L Erlenmeyer flasks to an absorbance of 0.6, and then induced with 1 mM isopropyl-ß-D-thiogalactoside (IPTG). After 16 h of growth cells were harvested by centrifugation at 3000 g for 10 min and suspended in 100 ml of 100 mM Tris-HCl buffer (pH 8.0).

### Purification of LecB and LecB-His6 by Affinity Chromatography

LecB and the His-tagged LecB were purified as described previously [15], [37]. In brief, bacterial cells were disrupted by freezing for at least 1 h at −20°C and subsequent sonication. The lysate was centrifuged at 10,000×g for 30 min, and the following steps were carried out at 37°C. The supernatant obtained after centrifugation was loaded onto a mannose agarose column (Sigma, volume 10 ml). After washing the column with 100 ml 100 mM Tris-HCl (pH 8.0), containing 150 mM NaCl, the bound protein was eluted with 20 ml of 20 mM D-mannose in 100 mM Tris-HCL (pH 8.0). The sample was concentrated by ultrafiltration using Vivaspin 20 microconcentrators (molecular weight cut-off: 5 kDa; Sartorius AG, Goettingen, Germany) and then washed with Millipore-pure water. The purified protein was stored at −20°C.

### Purification of OprF

OprF was purified to homogeneity from *P. aeruginosa* exactly as reported previously for *E. coli* (Brinkman *et al.*, 2000).

### Peroxidase Labeling of LecB

Peroxidase labeled LecB was prepared using glutaraldehyde coupling as described previously [38], [39], [40]: 1.5 mg of purified lectin and 3 mg of peroxidase (from horseradish, Sigma) were dissolved in 1 ml of 0.1 M phosphate buffer (pH 6.8) and mixed with 0.03 ml of a 1% glutaraldehyde solution for 3 h at room temperature. The mixture was then sequentially dialyzed against the same phosphate buffer for 2 h, against phosphate buffer containing 2 mg glycine/mL overnight, and several changes of phosphate-buffered saline (PBS) for 6 h. The labelled lectin sample was finally centrifuged at 30,000×g for 15 min to remove aggregates.

### Release of LecB from *P. aeruginosa* Cells by Washing with L-fucose

*P. aeruginosa* PAO1 containing plasmid pBBC2 was grown on NB agar plates at 37°C for 48 h. Bacterial cells (12 mg dry weight) were washed off with 20 ml PBS and then centrifuged for 10 min at 3,000×g. The cell pellet was washed three times in PBS and the cells were resuspended in PBS containing 20 mM L-fucose (Sigma) or 20 mM D-galactose (Sigma) as a negative control, and incubated for 1 h at 4°C. After centrifugation for 10 min at 3,000×g the sterile-filtered supernatant and an amount of the cell pellet equivalent to an OD at 580 nm of 0.15 were analyzed by Western blotting.

### Cell Fractionation of *P. aeruginosa* [23]

*P. aeruginosa* strains PAO1 and H636 containing plasmid pBBC2 and *P. aeruginosa* PAO1 without plasmid were grown on NB agar plates at 37°C for 48 h. Bacterial cells (1.2 mg dry weight) were washed off with 1 ml 0.14 M NaCl and then centrifuged at 3000 g; the supernatant was sterile-filtered and used to determine the amount of extracellular LecB. The cell pellet was carefully suspended in 240 µl 100 mM Tris-HCl (pH 8) containing 20 % (w/v) sucrose. After addition of 240 µl of the same buffer containing 5 mM EDTA and 20 µg lysozyme, the sample was incubated for 30 min at room temperature, spheroplasts were collected by centrifugation at 10.000 g for 20 min and the supernatant was used as the periplasmic fraction. Spheroplasts were disrupted by sonication (Sonifier W250; Branson) in 240 µl 100 mM Tris-HCl (pH 8). After centrifugation for 5 min at 5,000×g to remove undisrupted cells and cell debris, the total membrane fraction was collected by centrifugation for 45 min at 13,000×g and the supernatant was used as the cytoplasmic fraction. An amount equivalent to a cell density of an O.D._580_ nm of 0.5 of each fraction was used for Western blotting.

### Outer Membrane Isolation

Outer membranes were isolated by a modification of the method described previously [41]. *P. aeruginosa* PATI2 cells (500 mg dry weight) were harvested after growth for 48 h at 37°C by centrifugation at 3000 g for 10 min. The cells were resuspended in 200 ml 100 mM Tris-HCl (pH 8) containing 10 mg lysozyme, incubated for 30 min at 37°C and disrupted by three passages through a French press. Intact cells were separated from the cell extract by centrifugation at 5,000×g for 10 min. The supernatant was centrifuged at 13,000×g for 1 h. The pellet, consisting of the total membrane fraction, was resuspended in 10 ml 100 mM Tris-HCl (pH 8) containing 2 % lauryl sarcosinate and incubated at room temperature for 20 min. After centrifugation for 40 min to at 45.000 g the pellet consisting of the outer membrane fraction was resuspended in 100 mM Tris-HCl (pH 8.0).

### Isolation of LecB Ligands by Affinity Chromatography on D-mannose-agarose

*P. aeruginosa* PAO1 was grown in 0.5 l NB-medium at 37°C for 48 h. Bacterial cells were centrifuged at 3000 g for 10 min, the cell pellet was suspended in 20 ml 100 mM Tris-HCl and disrupted by freezing for at least 1 h at −20°C and subsequent sonication. The lysate was centrifuged at 10,000×g for 30 min, and the following steps were carried out at 37°C. Cleared cell extract was loaded on a mannose agarose column (Sigma, volume 5 ml). After washing the column with 30 ml 100 mM Tris-HCl (pH 8.0) containing 150 mM NaCl, the bound protein was eluted with 10 ml of 20 mM mannose in 100 mM Tris-HCL (pH 8.0). As a negative control, the same experiment was carried out with the *lecB*-deficient *P. aeruginosa* mutant PATI2. One ml each of the eluates was analyzed by SDS-PAGE, 2-D-gel electrophoresis and MALDI-TOF mass spectrometry.

### Isolation of LecB Ligands from the Outer Membrane

The isolation procedure was carried out at 37°C. The outer membrane fraction was incubated in 100 mM Tris-HCl containing 2 mg His-tagged LecB for 1 h. After loading the sample onto a Ni-NTA-agarose column (Quiagen, volume 5 ml)), the column was washed with 50 ml Tris-HCl (pH 8.0) containing 50 mM imidazole and 300 mM NaCl to remove non-specifically bound proteins. LecB binding proteins were eluted with 5 ml 100 mM Tris-HCl containing 20 mM L-fucose. 1 ml of the sample was analyzed by 2-D-gel electrophoresis and MALDI-TOF mass spectrometry.

### SDS-PAGE and 2 D Gel Electrophoresis

Prior to SDS-PAGE, samples were suspended in SDS-PAGE sample buffer, boiled for 5 min at 99°C and loaded onto an SDS-16% polyacrylamide gel. SDS-gel electrophoresis was run for 1 h at 200 V. For 2 D gel electrophoresis, the proteins were precipitated overnight with 20% (v/v) TCA and afterwards washed twice with acetone. The protein preparation was air dried and resuspended in 1 ml rehydration buffer (7 M urea, 2 M thiourea, 4% (w/v) 3-[(3-cholamidopropyl) dimethylammonio]-1-propanesulfonate (CHAPS), 2% IPG buffer and pH 3–11 negative-logarithmic stripes as recommended by the manufacturer (GE-Healthcare, Freiburg, Germany), 1% (v/v) bromphenol blue). Protein was loaded onto an IPG strip and isoelectric focusing was performed at a maximum voltage of 8,000 V. The second dimension SDS-gel electrophoresis was run for 3 h in a 12.5% polyacrylamide gel at 250 V. Afterwards, the gels were stained with Coomassie Brilliant Blue G250.

### Western Blotting

Proteins from 1-D-gels were electrophoretically transferred at 150 mA for 15 min, and at 300 mA for 20 min onto PVDF membranes (Bio-Rad). Electrophoretic transfer from 2-D-gels to PVDF membranes was performed by semi-dry blotting as described before [42]. The membranes were blocked with 3 % (w/v) BSA overnight at 4°C. LecB, EstA and DsbA were detected by incubating the membranes with specific polyclonal antibodies [43], [44], [45] at a dilution of 1∶20,000, 1∶85,000 and 1∶100,000 in TBST (25 mM Tris-HCl, pH 8, 150 mM NaCl, 3 mM KCl, 0.2% v/v Tween 20), respectively, followed by an anti-rabbit immunoglobulin G-horseradish peroxidase conjugate (Bio-Rad). The blots were developed with the ECL chemiluminescence kit (GE Healthcare). For detection of LecB ligands, the membranes were incubated either with 1 µg×ml^−1^ purified LecB protein in 10 mM TBS containing 3% bovine serum albumin (Fluka) 0.05% Tween 20 (ROTH) before exposure to the antibodies as described above or with 1 µg/ml peroxidase labelled LecB. The blots were developed with the ECL chemiluminescence kit (GE Healthcare).

### Glucose-6-phosphate Dehydrogenase Assay

Glucose-6-phosphate dehydrogenase was used as a cytoplasmic marker enzyme [8], [46]. A stock solution of NADP (45 mM) and a stock solution of glucose-6-phosphate (110 mM) were diluted 1∶100 in a buffer containing 55 mM Tris-HCl (pH 7.5) and 11 mM MgCl. A 900 µl volume of this test solution was mixed with 100 µl of a sample from cytoplasm, periplasm, membrane fraction and supernatant, respectively, and the decrease in optical density (OD340/min) was monitored spectrophotometrically at 30°C for 90 sec.

### Protein Identification by MALDI-MS

Spots of interest were excised from polyacrylamide gels and digested overnight with Trypsin Gold (Promega, USA) and eluted as described by Shevchenko *et al.* [*47*]. The diluted proteins were desalted if necessary with ZipTip C18 (Millipore, USA) and spotted on Prespotted AnchorChip (Bruker, Germany) with a HCCA (ά-cyano-4-hydroxycinnamic acid) matrix. The masses of the peptides were determined with an UltraflexIII system (Bruker, Germany). Database search was carried out witch MASCOT ([48]; www.matrixscience.com).

### Hemagglutination Assay

Rabbit red blood cells (Fiebig Nährstofftechnik, Germany) (RBC) were collected, washed three times in PBS and resuspended to a final concentration of 5 % (v/v). The erythrocyte suspension was diluted 9∶1 with PBS buffer containing papain (1 % (w/v) and L-cysteine (0.1 % (w/v)) and incubated for 1 h at 37°C. Afterwards, the suspension was washed three times in PBS and 50 µl were mixed with 50 µl of PBS containing *P. aeruginosa* cells. *P. aeruginosa* was grown on NB Agar for 48 h by 37°C. Bacterial cells were washed off with 1 ml 0.14 M NaCl and cells were isolated by centrifugation (10 min.; 3000×g) and the pellets washed three times with PBS and afterwards resuspended in 50 µl PBS. After incubation for 1 h at 37°C, the erythrocytes were sedimented by centrifugation (30 sec at 1000×g at room temperature) and the hemagglutination optically examined.

## Results

### LecB is Bound to the Surface of Biofilm Cells

**Figure 1:**
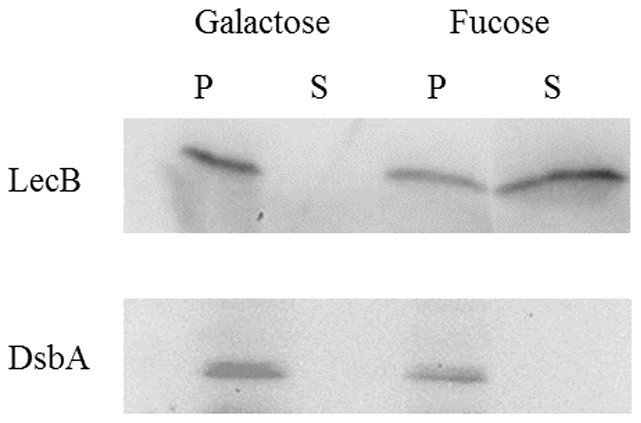
Release of LecB from the cell surface of *P. aeruginosa*. Biofilm cells were incubated with 20 mM L-fucose for 1 h at 4°C. Cell pellets (P) were separated from supernatants (S) by centrifugation and LecB was detected in both fractions by immunoblotting. Cells treated with 20 mM D-galactose served as a negative control. Additionally, blots were incubated with an antiserum against the periplasmic protein DsbA to monitor putative cell lysis during L-fucose treatment.

LecB can bind specific ligands located at the cell surface of *P. aeruginosa* [23] and biofilm formation can completely be inhibited by LecB specific fucosyl-peptide dendrimers [24]. These observations prompted us to investigate the location of LecB on the surface of *P. aeruginosa* cells grown as a biofilm. To this end, *P. aeruginosa* PAO1 harbouring plasmid pBBC2 which contains the wild-type *lecB* gene under the transcriptional control of a constitutive *lac* promoter was grown as a biofilm on the surface of NB agar for 48 h. Growing bacteria on leaf and food surfaces, as colonies, that have a continuous air-biofilm interface has been shown to result in the formation of unsaturated biofilms [3], [49], [50] of the type that is also found in the lungs of CF patients suffering from *P. aeruginosa* infections. Under these growth conditions, LecB is located in the bacterial outer membrane [23]. Cells were incubated with 20 mM of the high affinity ligand L-fucose at 4°C to release cell surface exposed LecB [14]. This low temperature was chosen to decrease the affinity of LecB for the ligands, since previous results had shown a minimal hemagglutination activity of LecB at 4°C [43]. Cells and supernatant were separated by centrifugation and analysed by SDS-PAGE and subsequent Western-blotting using antiserum directed against LecB [23] and DsbA [51], with the latter serving as a control to monitor whether cell lysis had occurred during fucose treatment. Fucose treatment led to the release of LecB, but not of DsbA into the supernatant, whereas cells treated with D-galactose did not release any LecB (Fig. 1). As expected, DsbA was detected only in the cell pellet fraction (Fig. 1).

### LecB Interacts with the Outer Membrane Porin OprF

**Figure 2:**
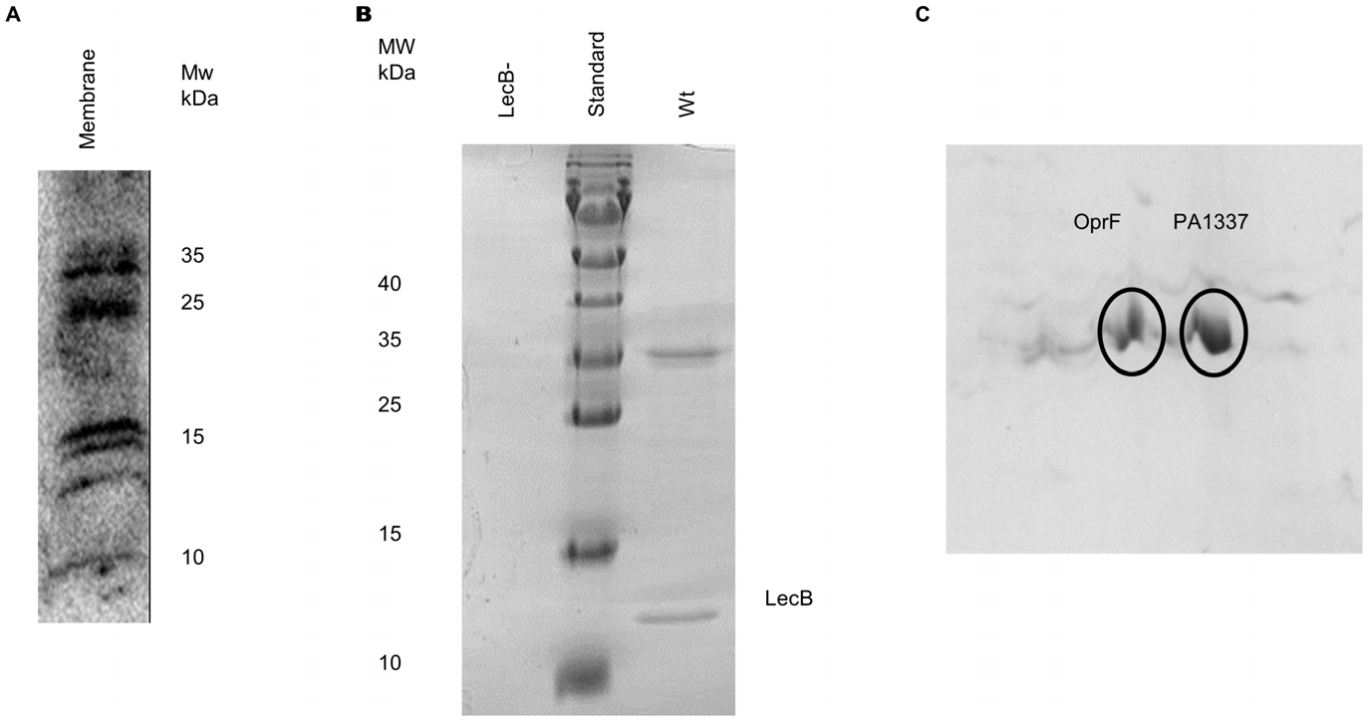
Identification of LecB interaction partners in *P. aeruginosa*. **A.** Far-Western blot analysis of membrane fractions from *P. aeruginosa* PAO1 using purified LecB and a LecB-specific antiserum. **B.** SDS-PAGE analysis of putative LecB ligands isolated from cell lysates of *P. aeruginosa* PAO1. Proteins were purified by affinity chromatography on mannose agarose, the column washed with 100 mM Tris-HCl (pH 8) and proteins eluted with 20 mM mannose in 100 mM Tris-HCl (pH 8) The *lecB* deficient mutant *P. aeruginosa* PATI2 served as a negative control. **C**.2D electrophoretic separation of protein bands shown in Fig. 2B; spots were subsequently analysed by MALDI-TOF mass spectrometry and identified as OprF and PA1337.

The finding that LecB could be released from the cell surface of *P. aeruginosa* encouraged us to search for putative LecB ligands. *P. aeruginosa* PAO1 was grown as an unsaturated biofilm on the surface of NB agar, the membrane fraction was isolated using differential cell fractionation and proteins were analysed by SDS-PAGE and subsequent Far-Western-blotting using purified LecB protein and a LecB-specific antiserum. Several immunoreactive bands could be identified in the membrane fraction representing putative LecB ligands (Fig. 2A).

Putative LecB ligands were purified by affinity chromatography on a mannose agarose matrix by utilizing the intrinsic specificity of LecB for D-mannose. In accordance with the purification protocol developed for LecB expressed in *E. coli* [15], [37], purification was carried out at 37°C. *P. aeruginosa* PAO1 cells grown in NB medium for 48 h were disrupted by sonication and the cell lysate was loaded onto a D-mannose agarose column. After washing the column, bound proteins were eluted with 100 mM Tris-buffer containing 20 mM D-mannose and analysed by SDS-PAGE (Fig. 2 B). As a negative control, the same experiment was performed using the *lecB* deficient mutant *P. aeruginosa* PATI2. LecB itself and two additional proteins with an apparent molecular mass in the range of 35 kDa were detected, further separated by two-dimensional gel electrophoresis (Fig. 2 C) and the resulting spots were identified by MALDI-TOF mass spectrometry as the outer membrane porin OprF (PA1777) (sequence coverage 48%, mascot score 328) and the putative glutaminase-asparaginase PA1337 (*ansB*) (sequence coverage 23%, mascot score 116). In contrast, cell lysates obtained from the *lecB*-negative strain *P. aeruginosa* PATI2 did not contain any proteins which could be isolated by affinity chromatography under these experimental conditions. Our findings clearly indicate that LecB interacts with the outer membrane porin OprF and the hypothetical protein PA1337. The interaction between LecB and OprF was further investigated by growing the *lecB*-deficient mutant *P. aeruginosa* PATI2 in NB medium for 48 h, isolation of the outer membrane and incubation with purified N-terminal His-tagged LecB. The preparation was loaded onto a Ni-NTA-agarose column to immobilize putative lectin-ligand complexes. After washing the column, proteins were eluted by washing with Tris-HCl buffer containing 20 mM L-fucose and eluted proteins were analysed by 2-DE (data not shown). A single spot was detected and the respective protein was identified by MALDI-TOF mass spectrometry as OprF. Elution of OprF upon addition of fucose indicated that the interaction of LecB and OprF was specific and further suggested that OprF itself may be glycosylated. A specific interaction between OprF and LecB was confirmed by transferring the eluted OprF to a blotting membrane and subsequent treatment with peroxidase-labelled LecB. Again, binding of LecB to OprF could be demonstrated (data not shown).

### LecB Binds OprF on the Cell Surface of *P. aeruginosa*

**Figure 3:**
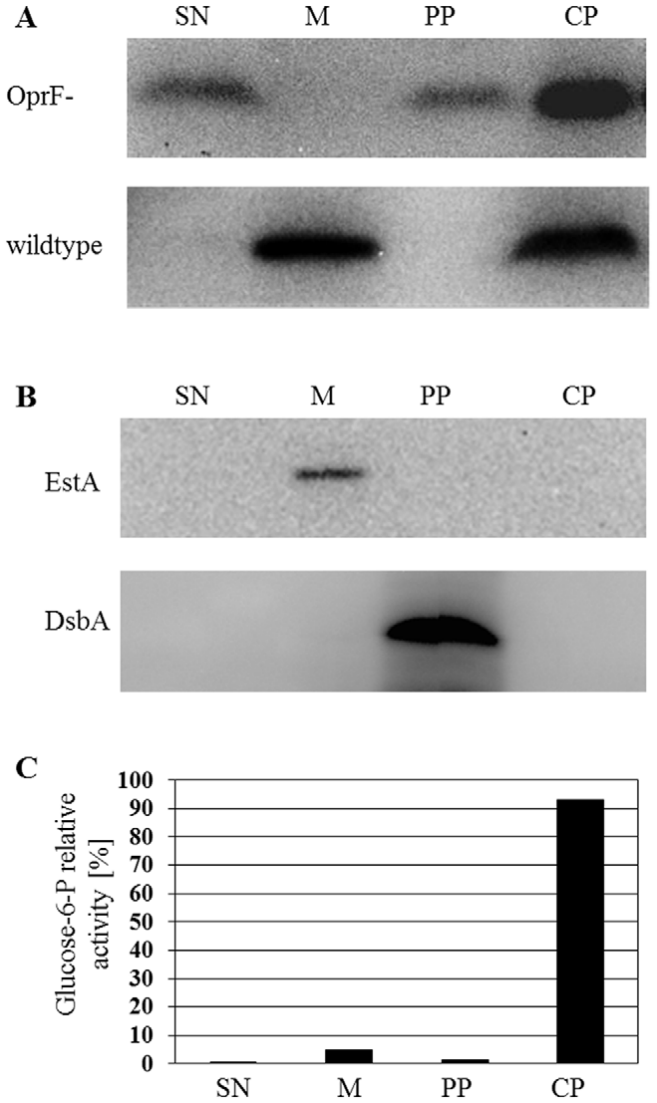
Subcellular localization of LecB in biofilm cells of the *oprF*-deficient mutant *P. aeruginosa* H636 grown for 48 h at 37°C. A. The same amount of culture supernatant, periplasm, cytoplasm and total membrane were subjected to SDS-PAGE analysis followed by immunoblotting using LecB antiserum. Fractions obtained from *P. aeruginosa* wild-type (wt) served as a positive control. Fractionation controls: **B.** Cellular fractions were analyzed using antisera directed against EstA (an outer membrane protein) and DsbA-(a periplasmic protein) and **C.** by determination of relative glucose-6-phosphate dehydrogenase (cytoplasmic protein) activities. The percentages of relative enzyme activities present in the cytoplasm (CP), the periplasm (PP), the membrane fraction (MF) and the culture supernatant (SN) are shown.

Apparently, LecB is cell surface exposed and interacts with OprF via carbohydrate ligands. We further investigated this interaction by growing the *oprF*-deficient *P. aeruginosa* mutant H636 which harboured plasmid pBBC2 containing the *lecB* gene as an unsaturated biofilm on the surface of NB agar. After differential cell fractionation proteins were analysed by SDS-PAGE and Western-blotting, the wild-type strain *P. aeruginosa* PAO1 served as a negative control. Interestingly, LecB was detected in the cytoplasm and in the periplasm as well as in the culture supernatant of the *oprF*-deficient strain, whereas in the wild-type strain, LecB was detected only in the cytoplasm and in the membrane fraction (Fig. 3). The release of LecB into the culture supernatant and its absence from the outer membrane fraction in the OprF deficient mutant strongly suggests a specific interaction between LecB and OprF in the outer membrane of *P. aeruginosa*.

### OprF is Needed for Hemagglutination Activity of *P. aeruginosa*

**Figure 4:**
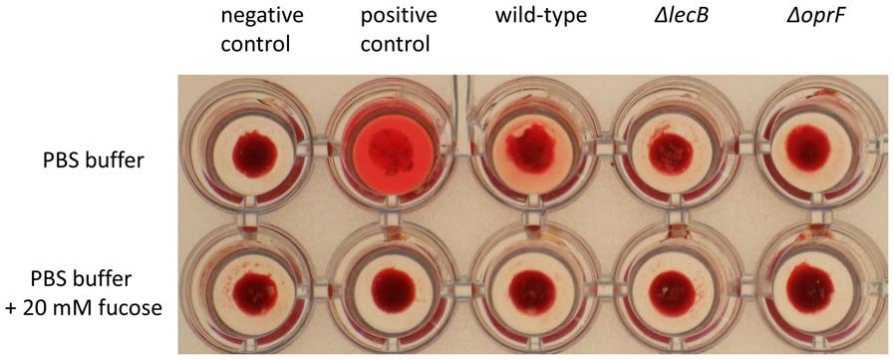
Hemagglutination of rabbit red blood cells after incubation with *P. aeruginosa*. Erythrocytes were treated with papain and L-cysteine and then incubated with either PBS buffer in the absence and presence of 20 mM fucose or PBS-buffer containing *P. aeruginosa* PAO1 wild-type and mutants Δ*lecB* and Δ*oprF*. The positive control additionally contained purified LecB protein (concentration: 10 µg ml^−1^). Non-agglutinated red blood cells form a distinct concentric pellet in the middle of the well whereas agglutinated red blood cells form a more extended and plaque-like structure.

Lectins mediate the agglutination of erythrocytes caused by *P. aeruginosa via* interaction with specific sugar molecules on the surface of blood cells [52]. Our results described above suggested that the deletion of the *oprF* gene may affect the hemagglutination activity of the respective *P. aeruginosa* mutant. To test this assumption, erythrocyte cells were incubated for 1 h with *P. aeruginosa* PAO1 wild type, *Δ*l*ecB* and *ΔoprF* mutants and agglutination was optically examined (Fig. 4). As expected, *P. aeruginosa* wild-type cells showed strong hemagglutination activity, whereas mutant strains *P. aeruginosa lecB* as well as *oprF* caused a significantly decreased agglutination. Purified LecB served as a positive and PBS buffer as a negative control. The agglutination caused by LecB as well as by wild-type *P. aeruginosa* cells could be inhibited by the addition of 20 mM L-fucose to the sample buffer. These results indicated that an interaction of the porin OprF and the lectin LecB in the outer membrane is required to mediate agglutination of red blood cells caused by *P. aeruginosa*.

## Discussion

*P. aeruginosa* is the major pathogen in the respiratory tract of patients suffering from cystic fibrosis. The treatment of these chronic *P. aeruginosa* airway infections is difficult due to the innate and adaptive antibiotic resistance of *P. aeruginosa* and the formation of biofilms on the respiratory epithelium [44], [53]. The lectin LecB and the major outer membrane porin OprF have both been shown to be involved in adhesion to lung epithelial cells [30], [54]. Our study now demonstrates that LecB is bound to the bacterial outer membrane and interacts with OprF. In an earlier study, we already showed that LecB is localized in the bacterial outer membrane of *P. aeruginosa* biofilm cells [23], but it remained unclear whether it faces the periplasm or the exterior. In this study, we washed biofilm cells with L-fucose which binds LecB with a high affinity (K_D_ = 1.5×10^6^ M^−1^) [14] and we observed the release of LecB from the cell surface. Further analysis of membrane fractions by Far-Western-blotting using purified LecB detected several putative LecB ligands. These results already indicated that LecB specifically interacts with glycoproteins present in the bacterial membrane. The major outer membrane porin OprF was then co-purified from wild-type *P. aeruginosa* using affinity chromatography on mannose agarose indicating an *in vivo* interaction of both proteins. The same method applied to a *lecB*-deficient mutant of *P. aeruginosa* did not result in isolation of OprF. Moreover, OprF could be isolated from the outer membrane fraction by His-tagged LecB immobilized on Ni-NTA agarose and could also be detected by affinity binding to peroxidase labelled LecB. Apparently, co-purification of OprF depended on specific binding to LecB which could be abrogated by subsequent washing of the column with the LecB-specific sugar fucose. Efficient *in vitro* binding of peroxidase labelled LecB to OprF blotted onto PVDF membranes after SDS-PAGE clearly suggested that LecB recognized OprF. So far, we failed to obtain any experimental evidence for glycosylation of OprF. Hence, the mechanism of the interaction between LecB and OprF remains unknown and requires further investigation.

Carbohydrate blood group antigens present on the surface of erythrocytes can bind to LecB and thereby cause hemagglutination. We have observed that a *P. aeruginosa lecB* deficient strain showed a significantly decreased hemagglutination activity as compared to the corresponding wild-type strain (Fig. 4). Interestingly, a *P. aeruginosa oprF* deletion mutant showed the same decrease in hemagglutination activity which could not be increased by expression of *lecB* from a plasmid. This result also strongly suggests an interaction of LecB with OprF on the cell surface of *P. aeruginosa*.

Interactions of lectins with cell surface proteins of pathogenic bacteria have been reported before [55]. Lectins located at the tip of pili or ﬂagella including PapG and GafD of uropathogenic *E. coli* are referred to as adhesins, as they play a role in adherence to epithelial cells [56]. In an earlier report, we demonstrated that LecB is an important factor in the development of biofilms by *P. aeruginosa* [23]. Furthermore, it was suggested that both LecB and OprF contribute to bacterial adherence to A549 epithelial cells [30], [54]. As *P. aeruginosa* is toxic to epithelial cells [57], promotion of adherence might manifest as increased cytotoxicity and consequent lung epithelial destruction. Therefore, it is tempting to speculate that LecB and OprF together may mediate adhesion of *P. aeruginosa* to receptors located on cells of either the same or of different species, thus enabling the colonization of host tissues as well as the formation of mono- or multispecies biofilms. Previously, it was reported that interferon gamma binds to OprF, resulting in the expression of another quorum-sensing dependent virulence determinant, the lectin LecA of *P. aeruginosa*. Interestingly, a fucosyl-residue is required for recognition of human interferon gamma by the receptor [58] suggesting that the fucose-specific LecB may act as an adaptor to mediate recognition of this cytokine by OprF on the bacterial surface. Thus, it would be interesting to test whether this regulatory effect on *lecA* expression through sensing of interferon gamma is still functional in a *lecB*-negative *P. aeruginosa* mutant.

In conclusion, our findings show that LecB and OprF are interaction partners *in vivo* but OprF is not involved in LecB secretion. Both proteins themselves have already been shown to influence key virulence-associated functions of *P. aeruginosa* [59]. Hence, the interaction of these proteins may modulate their individual functions and may even create novel functionalities affecting pathogenicity of *P. aeruginosa.*
